# A 204-subject multimodal neuroimaging dataset to study language processing

**DOI:** 10.1038/s41597-019-0020-y

**Published:** 2019-04-03

**Authors:** Jan-Mathijs Schoffelen, Robert Oostenveld, Nietzsche H. L. Lam, Julia Uddén, Annika Hultén, Peter Hagoort

**Affiliations:** 1 Radboud University, Donders Institute for Brain, Cognition and Behaviour, Nijmegen, The Netherlands; 2 NatMEG, Karolinska Institutet, Stockholm, Sweden; 3 Max Planck Institute for Psycholinguistics, Nijmegen, The Netherlands; 4 Stockholm University, Department of Psychology and Department of Linguistics, Stockholm, Sweden; 5 Swedish Collegium for Advanced Study, Uppsala, Sweden; 6 Department of Neuroscience and Biomedical Engineering, Aalto University, Espoo, Finland

**Keywords:** Electrophysiology, Psychology, Language, Functional magnetic resonance imaging

## Abstract

This dataset, colloquially known as the Mother Of Unification Studies (MOUS) dataset, contains multimodal neuroimaging data that has been acquired from 204 healthy human subjects. The neuroimaging protocol consisted of magnetic resonance imaging (MRI) to derive information at high spatial resolution about brain anatomy and structural connections, and functional data during task, and at rest. In addition, magnetoencephalography (MEG) was used to obtain high temporal resolution electrophysiological measurements during task, and at rest. All subjects performed a language task, during which they processed linguistic utterances that either consisted of normal or scrambled sentences. Half of the subjects were reading the stimuli, the other half listened to the stimuli. The resting state measurements consisted of 5 minutes eyes-open for the MEG and 7 minutes eyes-closed for fMRI. The neuroimaging data, as well as the information about the experimental events are shared according to the Brain Imaging Data Structure (BIDS) format. This unprecedented neuroimaging language data collection allows for the investigation of various aspects of the neurobiological correlates of language.

## Abstract (summary)

**Table:** 

Design Type(s)	behavioral data analysis objective • observation design
Measurement Type(s)	brain activity measurement
Technology Type(s)	magnetic resonance imaging • Magnetoencephalography
Factor Type(s)	biological sex • receptive language perception • Language
Sample Characteristic(s)	Homo sapiens • brain

Machine-accessible metadata file describing the reported data (ISA-Tab format)

## Background & Summary

A core aspect of understanding written or spoken language is the ability to combine the individual words of an incoming sentence into a cohesive message, specifying, among other things, who did what to whom. Sentence comprehension is the result of a dynamic contextual integration of individual word properties, relating to semantics and syntax, and goes far beyond mere concatenation of incoming words. The brain processes that underlie the cognitive operations that are required for adequate sentence comprehension are fundamental to the unique expressive power of human language.

The application of neuroimaging techniques to study the brain’s capacity for language has been an active area of research in the past few decades. Experimental studies have yielded a large body of information about the spatial and temporal dynamics of functional brain processes that support various aspects of language processing in general, and sentence processing in particular. Based on these experimental results, various neurobiological models have been put forward, linking specific aspects of language to their neurobiological underpinnings. There are various examples of such models^1–5^, but this list is by no means exhaustive. One specific model to mention is the Memory Unification and Control (MUC) model^6^, which gives an explicit account of how sentence processing might be realized at the level of the brain, by describing key processes that map onto functionally specialized cortical networks.

Measurement techniques that are often used in the study of the neurobiological underpinnings of language are functional magnetic resonance imaging (fMRI) and magnetoencephalography (MEG). Each of these techniques has its own specific spatial and temporal sensitivity, and complement each other with respect to the information that can be extracted from the data. The fMRI signal reflects fluctuations in the level of oxygenation of the blood, and provides high spatial resolution estimates of brain activity, but at low temporal resolution^7^. MEG, on the other hand, provides estimates of synaptic activity at a relatively low spatial resolution, but at high temporal precision^8^.

Typical experiments obtain data from a relatively limited number of subjects, and employ very specific and well-controlled experimental manipulations. The experimental effects are typically quantified and reported in terms of the averages across subjects, although this does not take into account the variability across individuals, which is known to be quite substantial^9,10^.

Furthermore, neuroimaging studies of language processing typically use one single sensory input modality for stimulus presentation (reading versus listening). From a conceptual point of view, however, it is often assumed that high-level aspects of language processing are independent of the stimulus modality.

The design of the current dataset aims to address some of the issues mentioned above. It contains neuroimaging data from a large set of 204 subjects, where from each subject we acquired both MEG and fMRI measurements, during which they performed the same language task. Furthermore, we acquired and included additional neuroimaging data: structural and diffusion-weighted data^11^ to image the anatomy and structural connections of the brain, as well as resting-state data from both imaging modalities. In the language task, subjects processed linguistic utterances, which either consisted of normal sentences or scrambled sentences. Half of the subjects were reading the stimuli, and the other half listened to the stimuli, allowing for a comparison between sensory input modality.

This dataset allows for the investigation of various aspects of the neurobiological correlates of language. Specifically, by virtue of the number of subjects, the multimodal nature of the data, and the two different sensory stimulation schemes used, it allows for studying (among others) in a principled way 1) individual differences in the brain’s response to language, 2) the relation between the brain response and the different neuroimaging modalities, and 3) the sensory-modality independent brain processes that facilitate sentence processing.

Compared to other public neuroimaging datasets such as the Human Connectome Project (HCP)^12,13^, the Cambridge Centre for Ageing and Neuroscience (CamCAN)^14^, and the Open MEG Archive (OMEGA)^15^ this dataset is the first dataset that contains both fMRI and MEG data for the full cohort of subjects, and contains longer than usual functional task recordings that are specifically dedicated to language processing.

The MEG has already been used in various peer-reviewed publications^16–19^. Publications using the fMRI data are in preparation. This *Data descriptor* is intended to comprehensively describe the experimental procedure and imaging protocol of the publicly released data, which is available at the Donders Institute’s data repository^20^. The datasets are owned by the Max Planck Institute for Psycholinguistics (Neurobiology of Language department).

## Methods

### Subjects

A total of 204 native speakers of Dutch (100 males) with a mean age of 22 years (range: 18 to 33 years) were included in the study. In the informed consent procedure, they explicitly consented for the anonymized collected data to be used for research purposes by other researchers. The subjects took part in both the fMRI and MEG part of the study, in a counterbalanced fashion. Each subject performed the task in either the visual or the auditory modality. All subjects were right-handed, had normal or corrected-to-normal vision, and reported no history of neurological, developmental or language deficits. The study was approved by the local ethics committee (CMO – the local “Committee on Research Involving Human Subjects” in the Arnhem-Nijmegen region) and followed guidelines of the Helsinki declaration.

### Stimulus material

The total stimulus set consisted of 360 sentences in Dutch, and their scrambled word list counterparts. The sentences consisted of two types: 180 of the sentences contained a relative clause (RC+), to create a more difficult syntactic structure. The other 180 sentences consisted of a main clause and a simple subordinate clause (RC−), to create an easier structure. The word lists were created by scrambling the words from the sentences such that three or more consecutive words did not form a coherent fragment. For an example of the sentences see Table 1. All sentences varied between 9 and 15 words in length.

**Table 1:** Exemplar sentences, word list and questions in Dutch, and literal English translation.

	Sentence	Word list
**Complex (Relative Clause, RC+)**	Het aardige vrouwtje gaf Henk die een kleurige **papegaai** gekocht had een zak pitjes *The nice lady gave Henk, who had bought a colorful parrot, a bag seeds*.	Zak een kleurige aardige een had die vrouwtje **papegaai** gaf het gekocht pitjes Henk *Bag a colorful nice a had who lady parrot gave the bought seeds Henk*
**Simple (RC−)**	Dit zijn geen regionale **problemen** zoals die op de Antillen. *These are no regional problems such as those on the Antilles*.	zoals geen die Antillen **problemen** regionale zijn de dit op *such as no those Antilles problems regional are the these on*

Relative clause sentences are more difficult to comprehend than simpler structures. Here, target words are presented in bold but were not marked in any manner during stimulus presentation.

Each subject was presented with a subset of 180 sentence stimuli, and 180 word list stimuli, where we ensured that, for a given sentence, they were not exposed to the corresponding word list. During the task MEG part of the experiment, the subjects were presented with 120 sentences and 120 word lists, and during the fMRI part of the experiment they were presented with 60 sentences and 60 word lists. Across subjects, all stimuli were presented the same number of times in the sentence and in the word list condition.

Each sentence and corresponding word list contained a noun that was at the same ordinal position, which varied between the third and thirteenth word position (which is denoted here as the ‘target word’). This allows for psychometrically controlled comparisons between the conditions using these isolated nouns. These words were matched in terms of lexical frequency and word length. To control for similarity in context, the word preceding the target did not differ in word length by more than two letters between a sentence and its word list counterpart. The word frequency was based on the lemma frequency according to the SUBTLEX-NL database of Dutch word frequencies^21^, and was on average 27,6/million (*SD* = 62,1/million). All stimulus material is shared along with the neuroimaging data.

The auditory versions of the stimuli were recorded by a female native Dutch speaker. The word lists were pronounced with a neutral prosody and a clear pause in between each word. The audio files were recorded in stereo at 44100 Hz. During the post processing the audio files were low-pass filtered at 8500 Hz and normalized such that all audio files had the same peak amplitude, and same peak intensity. In the word list condition, each word was separated by 300 ms of total silence. The transition from silence to speech was ramped at the onset (rise time of 10 ms) and offset (fall time of 10 ms) of single words in the word list condition, and for sentence onset. The onset of the first and target word vocalizations were determined manually for each audio file, using the Praat software (http://www.praat.org, RRID:SCR_016564)^22^.

### Experimental Design and Procedure

The total stimulus set was divided into two sets of 180, and each of these were subsequently divided into three subsets. Each subject was presented with 2/3 of the stimuli set in the MEG (120 trials of each condition) and 1/3 in the fMRI (60 trials). Across subjects, each subset was presented as many times in MEG as in fMRI. Different subjects that were presented with the same subset had the stimuli presented in a different (randomized) order. Within an experimental session, the stimuli were presented in blocks, alternating between sentence blocks (containing 5 sentences) and word list blocks (containing 5 word lists), for a total of 24 (MEG) or 12 (fMRI) blocks. The starting block type (either sentences or word list) was randomized across subjects.

In order to check for compliance, 20% of the trials were followed by a ‘Yes’/‘No’ question about the content of the previous sentence/word list. Half of the questions on the sentences addressed the content of the sentence (e.g. *Did grandma give a cookie to the girl*?) whereas the other half, and all of the questions about the word lists, addressed one of the main content words (e.g. *Was the word ‘grandma’ mentioned?*). A substantial part of the questions on complex relative clause (RC+) sentences concerned content from the relative clause. Subjects answered the question by pressing a button for ‘Yes’/‘No’ with their left index and middle finger, respectively.

At the start of each block there was a 1500 ms presentation of the block type: *zinnen* (sentences) or *woorden* (words). In sentences, the first word began with a capital letter, and the last word end with a full stop. The inter-trial interval was jittered between 3200–4200 ms. During this period, an empty screen was presented, followed by a fixation cross.

Stimuli were presented using the Presentation software (Version 16.0, Neurobehavioral Systems, Inc).

**Fig. 1:**
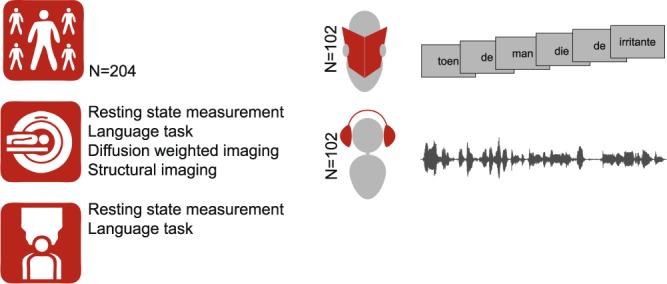
Schematic overview of the study procedure.

Prior to the task, subjects read a written instruction of the task and were allowed to ask questions for clarification. Furthermore, the experimenter emphasized that the sentences and word lists should be attended carefully, and discouraged attempts to integrate the words in the word list condition. Finally, to familiarize the subjects with the task, they did a practice task with stimuli separate from the actual study task. Figure 1 shows a schematic overview of the study procedure.

### Visual language task

The visual stimuli were presented with a LCD projector, with a vertical refresh rate of 60 Hz situated outside the MEG and fMRI scanning rooms, and projected via mirrors onto the screen inside the measurement room. All stimuli were presented in a black mono-spaced font on a gray background at the center of the screen within a visual angle of 4 degrees. Sentences or word lists were presented word-by-word with a mean duration of 351 ms for each word (minimum of 300 ms and maximum of 1400 ms, depending on word length). Specifically, the visual presentation rate of the stimuli was determined in relation to the duration of the audio recording of spoken versions of the sentences and the word lists (audiodur), taking into account both the number of letters (sumnletters) and words (nwords) in the whole sentence and the number of letters within each word (nletters). The duration of a single word (in ms) was determined as: (nletters/sumnletters) * (audiodur + 2000-150 * nwords). No word was presented for a shorter time than 300 ms. Each word was separated by an empty screen for 300 ms before the onset of the next word.

### Auditory language task

In the auditory task the stimuli were presented via plastic tubes and ear pieces to both ears. Before the experiment, the hearing threshold was determined individually and the stimuli were then presented at an intensity of 50 dB above the hearing threshold. In fMRI, the hearing threshold was determined on top of the EPI-sequence noise, to verify that all stimuli were clearly audible.

### Resting-state protocol

In each session (fMRI and MEG) resting-state recordings preceded the language task. In MEG, subjects were instructed to think of nothing specific while focusing on the fixation cross at the center of the screen for 5 minutes. The fixation cross was presented in the same format as in the visual task (sentence/word list). In fMRI, subjects were also instructed to think of nothing specific and not fall asleep while keeping their eyes closed for 7 minutes.

### MEG data acquisition

Magnetoencephalographic data were collected with a 275-channel axial gradiometer system (CTF). The signals were digitized at a sampling frequency of 1200 Hz (cutoff frequency of the analog anti-aliasing low pass filter was 300 Hz). Three head localizer coils were attached to the subject’s head (nasion, left and right ear canals) to determine the position of the head relative to the MEG-sensors. Throughout the measurement the head position was continuously monitored^23^. During breaks the subject was instructed to reposition and correct for small head position drifts if needed, and was therefore generally able to maintain a head position within 5 mm of the original position over the whole session. Three bipolar Ag/AgCl electrode pairs were used to measure the horizontal and vertical electro-oculogram, and the electrocardiogram. In the recordings where auditory stimuli were presented, the audio signals were recorded along with the MEG data using an ADC channel that was sampled synchronously with the MEG channels at 1200 Hz. We did not include an empty room recording in the MEG measurement protocol. Thus, in order to estimate the spatial structure of the noise, we recommend to estimate this (if needed) by means of independent component analysis for the resting-state data, or by using a well-defined baseline in the task data.

### MRI data acquisition

The data were acquired with a SIEMENS Trio 3 T scanner using a 32-channel head coil. The order of the different types of data was as follows: 1) resting-state fMRI, 2) task-based fMRI, pause, 3) structural image, 4) Diffusion-weighted imaging. From all subjects we also acquired MR-spectroscopy data, behavioral data, and genetics data, but this is not part of the released data set.

#### Task fMRI

During the task, we acquired T2*-weighted functional echo planar blood oxygenation level dependent (EPI-BOLD) data. We used a single echo 2D ascending slice acquisition sequence (with partial brain coverage) with the following specifications: Volume TR = 2.00 s, TE = 35 ms, 90 degree flip-angle, 29 oblique slices (position: L.076 A6.4 H16.6, orientation: T > C-5.8 > S-1.2, Phase encoding direction: A >> P, rotation: −0.80 degrees), slice-matrix size (base resolution) = 64 × 64, slice thickness = 3.0 mm, slice gap 0.5 mm, FOV = 224 mm, voxel size (x = FOV_x_/N_x_, y = FOV_y_/N_y_, z = slice thickness, anisotropic voxel size = 3.5 × 3.5 × 3.0 mm).

#### Resting state fMRI

T2*-weighted functional EPI-BOLD images (whole brain coverage) were acquired with a standard 2D gradient echo echo planar imaging sequence (TR = 1680 ms, TE = 30 ms, 70 degree flip-angle, slice-matrix = 64 × 64, FOV = 256 mm, anisotropic voxel size = 3.5 × 3.5 × 3.0 mm, slice orientation = I >> S, phase encoding direction: A >> P).

#### Structural imaging

A T1-weighted magnetization-prepared rapid gradient-echo (MP-RAGE) pulse sequence was used for the structural images, with the following parameters: volume TR = 2300 ms, TE = 3.03 ms, 8 degree flip-angle, 1 slab, slice-matrix size = 256 × 256, slice thickness = 1 mm, field of view = 256 mm, isotropic voxel-size = 1.0 × 1.0 × 1.0 mm. A vitamin-E capsule was placed as fiducial behind the right ear to allow a visual identification of left-right consistency.

#### Diffusion-weighted imaging

Diffusion weighted imaging (DWI) datasets consisted of 68 directions at b = 1000 s/mm^2^. Resolution was 2.2 × 2.2 × 2.2 mm; 64 slices were acquired with TR = 7700 ms, TE = 89 ms, matrix size 100 × 100 and GRAPPA acceleration factor 2.

### MEG coregistration procedure

In the MEG data, the locations of the MEG-sensors are defined relative to the subject’s head, where the subject’s specific coordinate system is defined based on a set of anatomical landmarks on the surface of the head (nasion, left and right pre-auricular points). This coordinate system was co-registered to the individual anatomical images, using the FieldTrip toolbox^24^. At the start of each MEG session, we recorded a digitized head surface, consisting of a set of x/y/z coordinates on the scalp, using a Polhemus 3D-Space Fastrak scanner. These points were expressed in the same coordinate system as the MEG sensor positions. Using a two-step procedure, the Polhemus head surface was aligned to the head surface extracted from the anatomical MRI. First, an approximate co-registration was performed by indicating the approximate location of the head localization coils in the anatomical MRI. The registration was refined using an automatic iterative closest point algorithm (https://nl.mathworks.com/matlabcentral/fileexchange/27804-iterative-closest-point), that aligned the Polhemus and the MRI head shape as close as possible. This was followed by visual inspection and if needed some manual adjustment. Manual adjustment was needed for some subjects due to the fact that the number of digitized surface points was not sufficiently large for the automatic ICP algorithm to find the optimal alignment. After this procedure, the median distance between the head surface points and the corresponding MRI head shape points was 1.97 mm (range 1.12-4.30 mm). The head surface points are part of the data distribution.

### BIDS data format conversion

Recently, community-wide efforts have resulted in the definition of a standard data representation of neuroimaging data (BIDS)^25,26^, in order to facilitate data sharing and scientific reproducibility. This standard imposes the data to be organized in a specific directory structure, using a well-defined file naming scheme and in standardized data formats. In addition, it specifies minimal requirements for accompanying “sidecar” text files with metadata in a both human- and computer-readable format. We adopted BIDS for the organization of the raw neuroimaging data that is presented here. The procedure for the conversion and reorganization of the data can be conceptually broken down into the following steps:

1. Creation of a directory structure according to BIDS
2. Collection and conversion of the MRI data from DICOM to NIfTI format
3. Collection and renaming of the MEG datasets in CTF format
4. Collection and adjustment of Presentation log files
5. Collection of the MEG co-registered anatomical MRIs
6. Creation of the sidecar files for each subject and defacing of anatomical MRIs
7. Creation of the general sidecar files
8. Validation and final refinements.

Steps 1–5 and 7 were implemented as shell scripts, and step 6 was implemented as a MATLAB script. The scripts used for the conversion can be found in the ‘code’ folder of the data repository. Here, we describe the relevant details of steps 2–4 and 6. The directory structure according to BIDS (step 1) will be described in more detail in the “Data records” section, and step 5 merely reflects the copying of data to another directory on the filesystem. The scripts used for the data format conversion are included in the data collection, and additional details on the procedure can be found online: www.fieldtriptoolbox.org/example/bids.

#### Collection and conversion the MRI data from dicom to NIfTI format

The original raw MRI data was in DICOM format. All images were converted into the NIfTI format, using the dcm2niix conversion software (http://www.nitrc.org/projects/dcm2nii/, RRID:SCR_014099). This conversion tool automatically created the MR-images’ relevant sidecar files in BIDS format, containing among others information about the MR acquisition parameters.

#### Collection and renaming of the MEG datasets

The raw MEG datasets were renamed using the CTF command line tool newDS. We used the ‘-anon’ option in order to anonymize the data. In addition to this step, we used a custom Bash shell script to scrub the acquisition date from the files.

#### Collection and adjustment of the Presentation log files

The Presentation log files are plain text files, containing details about the session-specific events and their timing (e.g. onset of stimuli, fMRI synchronization pulses, subject responses). Before including these files in the dataset for release, we used a custom Bash shell script to scrub the creation date and time from the file. The shell script was also used to correct some excess occurrences of newline characters in the log files; the Presentation scripts for the visual MEG stimulus task accidentally added the newline to the last word of each sentence.

#### Creation of the sidecar files for each dataset and defacing of anatomical MRIs

Besides the sidecar files that describe the details of the acquisition settings of the data, and the details of the channels acquired in the MEG session, we created events files for the fMRI and MEG data. These files contain, in a tab-separated textual format, a description of the events that occurred during data acquisition. For the fMRI data, these files were created from the Presentation log files, aligning the stimuli and responses to the time of the MR volumes, where time zero is defined as the start of acquisition. For the MEG data, information from the Presentation log files was combined with information from the trigger channels in the MEG data files.

The anatomical MRI data was de-identified by means of a defacing procedure based on the spm_deface function from SPM12 (http://www.fil.ion.ucl.ac.uk/spm/, RRID:SCR_007037).

The creation of the sidecar files, the aligning of the Presentation timing with MR acquisition and MEG triggers, and the defacing of anatomical MRI steps are implemented in the data2bids function, which is part of the FieldTrip toolbox^24^.

#### Creation of the general sidecar files

The general sidecar files, i.e. the subject and session specific scan.tsv files, as well as the collection specific participants.tsv and description file were created using functionality from the bids-tools toolbox, and supplemented by hand with textual information (the description file), and basic demographic information (sex and age in the participants.tsv file).

## Data Records

**Fig. 2:**
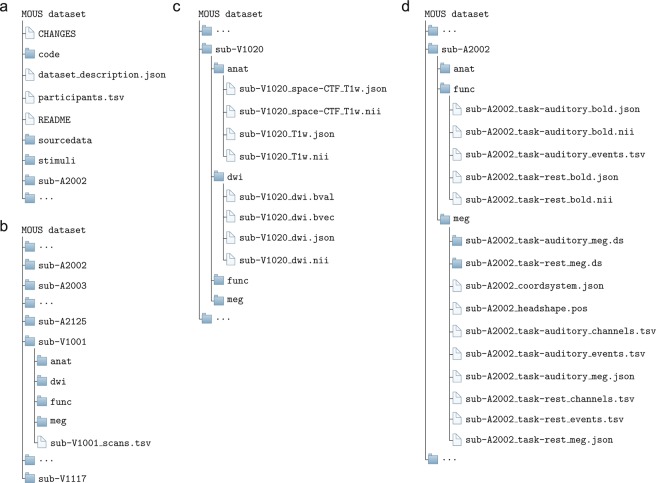
Organization of the data collection. (**a**) General overview of directory structure. (**b**) Content of subject specific directories. (**c**) Content of anatomy and diffusion weighted data directories. (**d**) Content of fMRI and MEG functional data directories.

The data collection can be accessed at the Donders Institute’s data repository^20^. The organization of the data collection is illustrated in Fig. 2. At the top-level (Fig. 2a) there is a small number of text files and folders containing the code to organize the data (in the ‘code’ folder), the original stimulus presentation log files (in the ‘sourcedata’ folder), the code used for stimulus presentation and the auditory wav-files (in the ‘stimuli’ folder), in addition to a separate folder for each of the subjects (Fig. 2b). The participants.tsv file lists the subject IDs along with sex and age, and is reproduced in Table 2. The subject naming scheme reflects the stimulus modality, where sub-A2* are subjects that listened to the stimuli, and sub-V1* are subjects that read the stimuli. The discontinuities in the numbering scheme reflects the dropout of some subjects, which was mainly caused by poor performance of those subjects on the control questions, failure to show up for the second data acquisition setting, or due to technical malfunction of lab equipment.

**Table 2:** List of subjects in the data collection with basic demographic information.

Participant_id	sex	age	Participant_id	sex	age	participant_id	sex	age	participant_id	sex	age
sub-A2002	F	25	sub-A2077	M	20	sub-V1001	M	20	sub-V1062	F	18
sub-A2003	F	23	sub-A2078	M	30	sub-V1002	F	20	sub-V1063	F	23
sub-A2004	F	21	sub-A2079	M	23	sub-V1003	M	24	sub-V1064	M	20
sub-A2005	F	21	sub-A2080	M	19	sub-V1004	F	22	sub-V1065	M	21
sub-A2006	F	22	sub-A2083	M	24	sub-V1005	F	21	sub-V1066	F	24
sub-A2007	F	21	sub-A2059	M	24	sub-V1006	M	22	sub-V1068	F	20
sub-A2008	F	18	sub-A2061	F	24	sub-V1007	F	21	sub-V1069	F	23
sub-A2009	F	22	sub-A2062	M	28	sub-V1008	F	25	sub-V1070	M	18
sub-A2010	F	24	sub-A2063	F	28	sub-V1009	F	23	sub-V1071	M	18
sub-A2011	M	18	sub-A2064	F	18	sub-V1010	F	20	sub-V1072	F	22
sub-A2013	M	21	sub-A2065	F	23	sub-V1011	F	21	sub-V1073	F	20
sub-A2014	M	20	sub-A2066	F	19	sub-V1012	F	20	sub-V1074	M	22
sub-A2015	F	19	sub-A2067	F	21	sub-V1013	F	21	sub-V1075	F	19
sub-A2016	F	18	sub-A2068	M	27	sub-V1015	F	24	sub-V1076	M	22
sub-A2017	F	28	sub-A2069	F	23	sub-V1016	F	21	sub-V1077	F	25
sub-A2019	F	29	sub-A2070	F	27	sub-V1017	F	18	sub-V1078	M	24
sub-A2020	M	22	sub-A2071	M	25	sub-V1019	M	20	sub-V1079	F	26
sub-A2021	F	19	sub-A2084	M	23	sub-V1020	M	22	sub-V1080	M	22
sub-A2024	F	24	sub-A2085	M	29	sub-V1022	F	19	sub-V1081	M	20
sub-A2025	F	22	sub-A2086	M	23	sub-V1024	F	19	sub-V1083	F	20
sub-A2027	F	21	sub-A2088	M	28	sub-V1025	M	19	sub-V1084	M	22
sub-A2028	M	26	sub-A2089	M	22	sub-V1026	M	19	sub-V1085	M	22
sub-A2029	F	20	sub-A2090	F	21	sub-V1027	F	23	sub-V1086	M	19
sub-A2030	F	23	sub-A2091	M	27	sub-V1028	F	21	sub-V1087	M	25
sub-A2031	F	20	sub-A2092	M	29	sub-V1029	F	23	sub-V1088	M	21
sub-A2032	F	25	sub-A2094	M	26	sub-V1030	F	28	sub-V1089	M	24
sub-A2033	F	27	sub-A2095	M	20	sub-V1031	F	23	sub-V1090	M	29
sub-A2034	M	28	sub-A2096	M	20	sub-V1032	M	26	sub-V1092	M	20
sub-A2035	M	19	sub-A2097	M	20	sub-V1033	M	19	sub-V1093	M	18
sub-A2036	M	19	sub-A2098	M	22	sub-V1034	M	21	sub-V1094	M	23
sub-A2037	F	20	sub-A2099	M	23	sub-V1035	M	23	sub-V1095	M	27
sub-A2038	M	19	sub-A2101	M	19	sub-V1036	F	24	sub-V1097	M	19
sub-A2039	F	19	sub-A2102	M	24	sub-V1037	F	18	sub-V1098	M	25
sub-A2040	F	20	sub-A2103	F	21	sub-V1038	M	20	sub-V1099	M	27
sub-A2041	M	21	sub-A2104	M	19	sub-V1039	F	21	sub-V1100	F	21
sub-A2042	F	20	sub-A2105	M	21	sub-V1040	M	29	sub-V1101	F	22
sub-A2046	F	20	sub-A2106	M	24	sub-V1042	M	25	sub-V1102	M	21
sub-A2047	F	26	sub-A2108	F	19	sub-V1044	M	23	sub-V1103	M	29
sub-A2049	F	21	sub-A2109	F	19	sub-V1045	F	20	sub-V1104	F	24
sub-A2050	M	21	sub-A2110	F	22	sub-V1046	F	21	sub-V1105	F	24
sub-A2051	M	24	sub-A2111	M	19	sub-V1048	F	19	sub-V1106	F	19
sub-A2052	F	21	sub-A2113	M	25	sub-V1049	F	18	sub-V1107	F	19
sub-A2053	M	23	sub-A2114	M	19	sub-V1050	F	19	sub-V1108	M	25
sub-A2055	M	24	sub-A2116	F	20	sub-V1052	M	20	sub-V1109	M	20
sub-A2056	F	19	sub-A2117	M	21	sub-V1053	M	20	sub-V1110	M	19
sub-A2057	M	21	sub-A2119	F	21	sub-V1054	F	19	sub-V1111	M	21
sub-A2058	F	22	sub-A2120	M	20	sub-V1055	F	22	sub-V1113	M	32
sub-A2072	F	20	sub-A2121	F	19	sub-V1057	F	21	sub-V1114	M	22
sub-A2073	M	23	sub-A2122	F	23	sub-V1058	F	20	sub-V1115	F	22
sub-A2075	M	27	sub-A2124	F	21	sub-V1059	M	19	sub-V1116	F	23
sub-A2076	M	21	sub-A2125	F	19	sub-V1061	M	23	sub-V1117	F	20

The ‘stimuli’ folder contains a text file listing the stimuli used for the language task. The sentences and word lists are indexed, where the indices relate to the correspondingly indexed wav-files in the ‘audio_files’ subfolder. The ‘presentation_code’ subfolder contains the stimulus presentation code used for the language task, separately for the different imaging and sensory stimulation modalities. We used 6 different scenarios, consisting different sets of stimuli to be presented to the subjects. These different scenarios are reflected in the numbered text files in the respective subfolders, which contain information about the specific stimuli used.

The ‘sourcedata’ folder contains for each of the imaging modalities for each of the subjects the original stimulus presentation log files. The numbering in the naming scheme allows to identify the specific scenario that the subjects have been exposed to.

Each of the subjects’ folder contains 4 subfolders, named ‘anat’, ‘dwi’, ‘func’ and ‘meg’, containing the anatomical, diffusion weighted and functional MRI, and the MEG data, respectively (Fig. 2b). In the next sections we describe in more detail the contents of these folders.

### Anat folder

The ‘anat’ folder (Fig. 2c) contains two NIfTI files with the 3-D anatomical data. Facial features were removed from the data. The ‘sub-#_T1w.nii’ file contains the anatomy in the MR scanner coordinate system. The ‘sub-#_space-CTF_T1w.nii’ file contains the anatomy following realignment to the MEG-based coordinate system that is linked to the anatomical landmarks. The MEG co-registration information (voxel-to-head coordinate transformation matrix) has been created by a manual co-registration procedure, identifying anatomical landmarks and fiducial locations, using the ‘ft_volumerealign’ function from FieldTrip. The file names containing ‘space-CTF’ are not valid BIDS names and therefore have been included in the dataset’s. bidsignore file to avoid issues with the BIDS validator (see technical validation section).

The sidecar json-files are text files which contain information about the MR acquisition parameters.

### DWI folder

The ‘dwi’ folder (Fig. 2c) contains the diffusion-weighted imaging data, as a 4D NIfTI file with its corresponding sidecar json-file. In addition, there are two text files, bval and bvec, containing information about the direction gradients. The sidecar json-file contains information about the MR acquisition parameters.

### Func folder

The ‘func’ folder contains the functional MRI data, for the language task and resting state measurements, as 4D NIfTI files, paired with their corresponding json-files (Fig. 2d). The language task data also has a corresponding events.tsv file, which provides information about the timing of experimentally relevant events. The sidecar json-file contains information about the MR acquisition parameters.

### MEG folder

The ‘meg’ folder contains the MEG data, for the language task and the resting state measurements. In line with the BIDS standard, the data are represented in the CTF specific format. This means that each of the tasks is in a separate folder with the extension ‘*.ds’, containing the binary data and a set of auxiliary files. In addition, each of the ‘*.ds’ folders has a set of three accompanying sidecar files: 1) a _channels.tsv file which lists the channels present in the data, 2) a _meg.json file with information about the acquisition parameters, and 3) an _events.tsv file with information about the timing of experimentally relevant events.

Next to this, the _headshape.pos file contains a set of digitized head surface points that was acquired with the Polhemus and used for the coregistration, and the _coordsystem.json file contains information about the coordinate system in which the location of the MEG sensors, the landmarks and digitized head surface points, and the head localizer coils are expressed.

## Technical Validation

### MEG

All subjects were monitored during data acquisition to ensure task compliance and general data quality (head movement, eye movements). Signal quality of the MEG was monitored online (muscle and eye movement artifacts), and subjects were given feedback for repositioning, if needed.

Despite the fact that we took the utmost care to generate a fully consistent dataset, a small number of the data records have some issues, which are listed in the ‘Data Usage’ section below. These issues were caused by a variety of factors, including technical failure, subject non-compliance and human error. We do not think that those issues limit the usability of the data.

MEG data were visually inspected for eye blinks/movements, muscle artifacts and jump artifacts, and their occurrences were marked. The result of the MEG to anatomy co-registration procedure was visualized and inspected.

### Task fMRI

For 11 subjects (3 from the visual, 8 from the auditory sample), the phase encoding direction was unintentionally left to right. Visual inspection of the data revealed minor signal dropout for 21 subjects (5 from visual, 16 from auditory). Movement and rotation were checked for each individual subject. For one subject (sub-V1004) movement exceeded 4 mm (4.5 mm), for this subject a sufficient number of volumes was unaffected by movement to allow for a meaningful estimation of a first-level statistical general linear model, so we did include this subject in the dataset.

### Resting state fMRI

For 9 subjects (1 from the visual, 8 from the auditory sample), the phase encoding direction was unintentionally left to right.

**Fig. 3:**
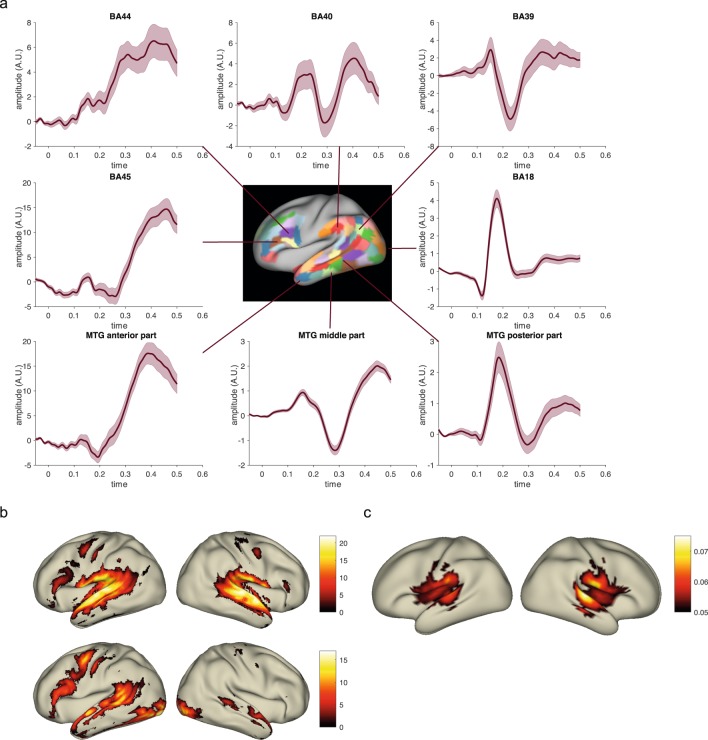
Group-level results obtained from the data. (**a**) MEG source-level event-related response to visual word onset for a set of highlighted cortical parcels (mean +/− SEM, n = 102, minimum norm estimate). BA = Brodmann Area, MTG = middle temporal gyrus. (**b**) fMRI statistical parametric maps for the contrast activation versus baseline, thresholded at a T-value of 0, for the auditory subjects (upper panels: n = 102) and for the visual subjects (lower panels: n = 102). (**c**) MEG source-level maps of delta frequency (1–3 Hz) entrainment of the brain activity to the acoustic envelope (n = 102, beamformer based coherence estimation).

To give an impression of the data quality Fig. 3 shows some basic group-level results for the MEG and fMRI task data. After the conversion of the data collection to the format according to BIDS, we used the BIDS-validator to ensure that the data was formatted properly according to this standard. Using version 1.2.2, the validator ran mostly without errors. The one remaining content related error pertained to a known issue for the validation of BIDS data containing CTF MEG data, which has been fixed in a newer version of the validator. In this version of the data collection we decided to exclude the CTF coregistered anatomical images from validation, by including these files in a.bidsignore file, rather than including these anatomical files in a derivatives folder.

## Usage Notes

The data collection is available at the Donders Institute’s data repository^20^. In line with the informed consent obtained from the subjects, the requirements of the Ethics Committee and of the Radboud University security officer, potentially identifying data (such as imaging data) recorded at the Donders Centre for Cognitive Neuroimaging can only be shared to researchers following explicit approval of a Data Use Agreement (DUA), hence the requirement for registration and requesting access. Neither the authors nor the data manager is involved in granting access to specific external researchers, this is only based on the complete registration of the researcher and follows a “click-through” procedure. Access to the data repository requires a login that can be based on an ORCID account. After agreeing to the DUA, the files in the collection can be accessed and downloaded in partial or full.

### Structure and interpretation of the events.tsv files

The _events.tsv are ASCII tab-separated values files with five columns for each of the events that occurred during the experimental measurement. The MEG language task associated events files have been constructed by combining event information extracted from the digital trigger channels in the MEG data with information from the stimulus presentation log files. As a consequence, in these files, the majority of events are duplicated.

The MEG resting state associated events files have been constructed from the MEG header information. The functional MRI language task associated events files have been constructed from the stimulus presentation log files alone. The resting state MRI data does not have an associated events file.

Each row in the events file reflects an event of a specific type and value, it has an onset time expressed in seconds relative to the onset of the data acquisition, a timestamp relative to the onset of the data acquisition (expressed in samples for the MEG, and in volumes for the fMRI), and a duration.

The event types ‘Sound’ and ‘Picture’, which have been derived from the stimulus presentation log files are text strings, and often contain one or two numeric values. These values map onto the corresponding digital trigger in the MEG data, reflect the stimulus wav-file that was presented (for the auditory task data), the identity of the question asked (all data), or reflected the intended duration for the respective word picture (for the visual task data).

Tables 3–6 illustrate the content of the different types of events files, and Table 7 shows the meaning of the numeric event values.

**Table 3:** Contents of _events.tsv file for the MEG auditory task data.

MEG task-auditory		
*type*	*origin*	*interpretation*
Nothing	presentation log file	-onset time of first word -onset of ‘target’ word -end of wav-file
Pause	presentation log file	onset of a pause between blocks
Picture	presentation log file	-onset of fixation cross on the screen with intended duration. -onset of question with identity of the question, matching the question in the stimuli presentation code. -onset of mini-block condition -onset of a blank screen
Quit	presentation log file	final event, when experimenter pressed the ‘quit’-button on the presentation PC.
Response	presentation log file	button press by subject
Resume	presentation log file	experiment resumes after pause
Sound	presentation log file	start of an audio wav-file. The value contains the identity of the wav-file used. This corresponds to the files in the stimuli folder of the data collection
UPPT001	MEG trigger channel	event logged by digital trigger channel. Integer value reflects the nature of the event (see Table 7)
UPPT002	MEG response channel	button press by subject
frontpanel trigger	MEG trigger channel	irrelevant historic remnant
trial	MEG data header	irrelevant historic remnant

**Table 4:** Contents of _events.tsv file for the MEG visual task data.

MEG task-visual		
*type*	*origin*	*interpretation*
Pause	presentation log file	onset of a pause between blocks
Picture	presentation log file	-onset of fixation cross on the screen with intended duration. -onset of question with identity of the question, matching the question in the stimuli presentation code. -onset of mini-block condition -onset of a blank screen -onset of individual word, including the word identity, the corresponding trigger value, and the intended length in milliseconds -onset of inter-stimulus interval
Response	presentation log file	button press by subject
Resume	presentation log file	experiment resumes after pause
UPPT001	MEG trigger channel	event logged by digital trigger channel. Integer value reflects the nature of the event (see Table 7)
UPPT002	MEG response channel	button press by subject
frontpanel trigger	MEG trigger channel	irrelevant historic remnant
trial	MEG data header	irrelevant historic remnant

**Table 5:** Contents of _events.tsv file for the MRI auditory task data.

fMRI task-auditory		
*type*	*origin*	*interpretation*
Nothing	presentation log file	-onset time of first word -onset of ‘target’ word -end of wav-file
Picture	presentation log file	-onset of fixation cross on the screen with intended duration. -onset of question with identity of the question, matching the question in the stimuli presentation code. -onset of mini-block condition -onset of a blank screen
Pulse	presentation log file	synchronization pulse for fMRI acquisition
Response	presentation log file	button press by subject
Sound	presentation log file	start of an audio wav-file. The event value contains the identity of the wav-file used. This corresponds to the files in the stimuli folder of the data collection

**Table 6:** Contents of _events.tsv file for the MRI visual task data.

fMRI task-visual		
*type*	*origin*	*interpretation*
Pause	presentation log file	onset of a pause between blocks
Picture	presentation log file	-onset of fixation cross on the screen with intended duration. -onset of question with identity of the question, matching the question in the stimuli presentation code. -onset of mini-block condition -onset of a blank screen -onset of individual word, including the word identity, the corresponding trigger value, and the intended length in milliseconds -onset of inter-stimulus interval
Pulse	presentation log file	synchronization pulse for fMRI acquisition
Response	presentation log file	button press by subject

**Table 7:** Explanation of trigger values as present in the MEG digital trigger channel, and as numeric values in the stimulus presentation log files.

Trigger value	meaning
1	Onset of individual word (visual task) or first word (auditory task) in a Relative Clause containing sentence (RC+).
2	Onset of ‘target’ word in a RC+ sentence.
3	Onset of individual word (visual task) or first word (auditory task) in a word list derived from a RC+ sentence.
4	Onset of ‘target’ word in a word list derived from a RC+ sentence.
5	Onset of individual word (visual task) or first word (auditory task) in a sentence without a relative clause (RC−).
6	Onset of ‘target’ word in a RC− sentence.
7	Onset of individual word (visual task) or first word (auditory task) in a word list derived from a RC− sentence.
8	Onset of ‘target’ word in a word list derived from a RC− sentence.
10	Mini block instruction stimulus ‘WOORDEN’ (words) or ‘ZINNEN’ (sentences)
11	Response (index), auditory task (in visual task, this event has value 1)
12	Response (middle), auditory task (in visual task, this event has value 2)
13	Experimenter response to continue after break, auditory task (in visual task, this event has value 3)
14	Start of audio file (auditory task only)
15	Offset of word picture (visual task) or audio file (auditory task)
20	Fixation picture, pre-trial baseline.
30	Pause
40	Question

### Additional notes about the MEG data

- There is a known fixed delay between the event timing and the actual timing of visual presentation of stimuli to the subject of 36 milliseconds.
- There is a variable delay (both across trials and across subjects) between the event timing (onset of the wav file) and the actual timing of auditory presentation of stimuli to the subject. This delay is on the order of slightly more than 60 milliseconds. In principle, detailed temporal alignment can be achieved comparing the audio traces in the MEG data with the corresponding stimulus wav-files.
- Interpretation of additional channels in the MEG data:
  - EEG057: bipolar vertical EOG channel
  - EEG058: bipolar horizontal EOG channel
  - EEG059: bipolar ECG channel
  - UADC003/UADC004: analog input channel with audio signal (auditory subjects only).

### Additional note about the task behavioral data

The subjects’ performance on the probe questions is not represented as such in the events.tsv files. The original stimulus presentation log files in the sourcedata folder contain this information. To assess the subject’s behavioral performance on the questions, one can count the number of occurrences of ‘hits’ and ‘incorrects’ in the ‘stim type’ column of the presentation log files.

### Known exceptions and issues

- Missing resting state data: For a small set of subjects we did not manage to obtain resting state MEG (sub-V1001, sub-V1002, sub-V1003, sub-V1005, and sub-A2119), or resting state fMRI (sub-V1025).
- MEG task data consisting of more than 1 run: For a small set of subjects, due to the acquisition software crashing upon data collection, the MEG task data is split into 2 data files. These datasets are named sub-#_task-#_run-1_meg.ds and sub-#_task-#_run-2_meg.ds for the following subjects: sub-A2011, sub-A2036, sub-A2062, sub-A2063, sub-A2076, sub-A2084, sub-V1006, sub-V1090.
- MEG task data from 2 different sets of stimuli: For subject sub-A2036, the acquisition software crashed, and the stimulus presentation was accidentally stopped in the process. Due to limitations in the presentation software, the same experimental scenario could not be continued, and a new scenario was started, using a different set of stimuli.
- MEG digital trigger issue 1: In a small set of auditory subjects, there was an issue with the digital trigger channel, where some of the bits of the triggers were sent in ‘level mode’, rather than in ‘pulse mode’. As a consequence, automatic identification of triggers from the MEG digital trigger channel might lead to incorrect trigger values. Therefore, not all UPPT001 digital trigger values are correct, and a small number show a mismatch with the corresponding event recovered from the stimulus presentation log file. This issue affects subjects sub-A2036, sub-A2037, sub-A2039, sub-A2050. The events coded in the corresponding _events.tsv file should be used instead of the UPPT001 trigger channel.
- MEG digital trigger issue 2: In a number of auditory subjects, the pulse width for the digital triggers to be sent by the stimulus presentation software was too wide (>30 ms), causing occasional ‘staircase’ triggers (if two events were too closely spaced in time) in the digital trigger channel. As a consequence, not all UPPT001 digital trigger values are correct, and a small number show a mismatch with the corresponding event recovered from the stimulus presentation log file. The affected subjects are listed in Table 8. The events coded in the corresponding _events.tsv file should be used instead of the UPPT001 trigger channel.

**Table 8:** List of subjects with digital trigger pulse width of >30 ms.

sub-A2014	sub-A2037	sub-A2047	sub-A2056
sub-A2029	sub-A2039	sub-A2049	sub-A2057
sub-A2031	sub-A2040	sub-A2050	sub-A2059
sub-A2033	sub-A2041	sub-A2051	sub-A2064
sub-A2035	sub-A2042	sub-A2053	sub-A2066
sub-A2036	sub-A2046	sub-A2055
- MEG incomplete task data: Due to time constraints in the lab and technical issues, the MEG auditory task data is incomplete for subjects sub-A2009 (first 219 trials out of 240), and sub-A2116 (first 225 trials out of 240). For sub-A2002 the first 20 trials (out of 240) have not been recorded.
- fMRI phase encoding direction: For 12 subjects, (3 from the visual, 9 from the auditory sample), the phase encoding direction was unintentionally left to right. The affected subjects are listed in Table 9.

**Table 9:** List of subjects with fMRI phase encoding direction from left to right.

sub-A2007 (rest)	sub-A2083 (task + rest)	sub-A2113 (task)
sub-A2032 (task)	sub-A2092 (task + rest)	sub-V1019 (task + rest)
sub-A2062 (rest)	sub-A2103 (task + rest)	sub-V1076 (task)
sub-A2073 (task + rest)	sub-A2110 (task + rest)	sub-V1092 (task)

## ISA-Tab metadata file

Download metadata file

## Data Availability

The custom written code (shell scripts and MATLAB scripts to implement steps 1-to organize the data according to the BIDS standard is included in the data release. The data2bids MATLAB-function is part of FieldTrip, and is available from https://github.com/fieldtrip/fieldtrip, the bids-tools are available from https://github.com/robertoostenveld/bids-tools, the bids validator is available from https://github.com/bids-standard/bids-validator, and the DICOM to NIfTI converter is available from https://github.com/rordenlab/dcm2niix.

## References

[1] Hickok G, Poeppel D (2007). The cortical organization of speech processing. Nat. Rev. Neurosci..

[2] Binder JR, Desai RH (2011). The neurobiology of semantic memory. Trends Cogn. Sci..

[3] Friederici AD (2012). The cortical language circuit: from auditory perception to sentence comprehension. Trends Cogn. Sci..

[4] Fedorenko E, Thompson-Schill SL (2014). Reworking the language network. Trends Cogn. Sci..

[5] Hagoort P, Indefrey P (2014). The neurobiology of language beyond single words. Annu. Rev. Neurosci..

[6] Hagoort P (2013). MUC (Memory, Unification, Control) and beyond. Front. Psychol.

[7] Menon RS, Kim SG (1999). Spatial and temporal limits in cognitive neuroimaging with fMRI. Trends Cogn. Sci..

[8] Hämäläinen M, Hari R, Ilmoniemi RJ, Knuutila J, Lounasmaa OV (1993). Magnetoencephalography—theory, instrumentation, and applications to noninvasive studies of the working human brain. Rev. Mod. Phys..

[9] Seghier ML, Price CJ (2018). Interpreting and Utilising Intersubject Variability in Brain Function. Trends Cogn. Sci..

[10] Fedorenko E, Kanwisher N (2009). Neuroimaging of Language: Why Hasn’t a Clearer Picture Emerged?. Lang. Linguist. Compass.

[11] Mori S, Zhang J (2006). Principles of diffusion tensor imaging and its applications to basic neuroscience research. Neuron.

[12] Larson-Prior LJ (2013). Adding dynamics to the Human Connectome Project with MEG. Neuroimage.

[13] Van Essen DC (2013). The WU-Minn Human Connectome Project: an overview. Neuroimage.

[14] Taylor JR (2017). The Cambridge Centre for Ageing and Neuroscience (Cam-CAN) data repository: Structural and functional MRI, MEG, and cognitive data from a cross-sectional adult lifespan sample. Neuroimage.

[15] Niso G (2016). OMEGA: The Open MEG Archive. Neuroimage.

[16] Schoffelen J-M (2017). Frequency-specific directed interactions in the human brain network for language. Proc. Natl. Acad. Sci. USA.

[17] Lam NHL, Schoffelen J-M, Uddén J, Hultén A, Hagoort P (2016). Neural activity during sentence processing as reflected in theta, alpha, beta, and gamma oscillations. Neuroimage.

[18] Lam NHL, Hultén A, Hagoort P, Schoffelen J-M (2018). Robust neuronal oscillatory entrainment to speech displays individual variation in lateralisation. Language, Cognition and Neuroscience.

[19] Hultén A, Schoffelen J-M, Uddén J, Lam NHL, Hagoort P (2019). How the brain makes sense beyond the processing of single words - An MEG study. Neuroimage.

[20] Schoffelen, J. M. *et al*. Mother of unification studies, a 204-subject multimodal neuroimaging dataset to study language processing. *Donders Repository*. http://hdl.handle.net/11633/di.dccn.DSC_3011020.09_236 (2019). 10.1038/s41597-019-0020-y PMC6472396 30944338

[21] Keuleers, E., Brysbaert, M. & New, B. SUBTLEX-NL: a new measure for Dutch word frequency based on film subtitles. *Behav. Res. Methods* **42**, 643–650 (2010). 10.3758/BRM.42.3.643 20805586

[22] Boersma, P. & Weenink, D. Praat: doing phonetics by computer. *Glot international* **5**, 341–345 (2001).

[23] Stolk A, Todorovic A, Schoffelen J-M, Oostenveld R (2013). Online and offline tools for head movement compensation in MEG. Neuroimage.

[24] Oostenveld R, Fries P, Maris E, Schoffelen J-M (2011). FieldTrip: Open source software for advanced analysis of MEG, EEG, and invasive electrophysiological data. Comput. Intell. Neurosci.

[25] Gorgolewski KJ (2016). The brain imaging data structure, a format for organizing and describing outputs of neuroimaging experiments. Sci. Data.

[26] Niso G (2018). MEG-BIDS, the brain imaging data structure extended to magnetoencephalography. Sci. Data.

