# Publisher Correction: A 204-subject multimodal neuroimaging dataset to study language processing

**DOI:** 10.1038/s41597-019-0050-5

**Published:** 2019-04-26

**Authors:** Jan-Mathijs Schoffelen, Robert Oostenveld, Nietzsche H. L. Lam, Julia Uddén, Annika Hultén, Peter Hagoort

**Affiliations:** 1 Radboud University, Donders Institute for Brain, Cognition and Behaviour, Nijmegen, The Netherlands; 2 NatMEG, Karolinska Institutet, Stockholm, Sweden; 3 Max Planck Institute for Psycholinguistics, Nijmegen, The Netherlands; 4 Stockholm University, Department of Psychology and Department of Linguistics, Stockholm, Sweden; 5 Swedish Collegium for Advanced Study, Uppsala, Sweden; 6 Department of Neuroscience and Biomedical Engineering, Aalto University, Espoo, Finland

Correction to: *Scientific Data* 10.1038/s41597-019-0020-y, published online 03 April 2019

In the original version of this Data Descriptor, the author Annika Hultén was listed incorrectly as being affiliated with *NatMEG*, *Karolinska Institutet*, *Stockholm*, *Sweden*. This has been corrected in both the HTML and PDF versions to *Max Planck Institute for Psycholinguistics*, *Nijmegen*, *The Netherlands*.

